# Unexplained visual loss after primary pars-plana-vitrectomy with silicone oil tamponade in fovea-sparing retinal detachment

**DOI:** 10.1186/s12886-023-02823-6

**Published:** 2023-02-24

**Authors:** T. Barth, H. Helbig, D. Maerker, M.-A. Gamulescu, V. Radeck

**Affiliations:** Department of Ophthalmology, University Medical Centre Regensburg, Franz-Josef-Strauß-Allee 11, 93053 Regensburg, Germany

**Keywords:** Silicone oil tamponade, Retinal detachment, Unexplained visual loss, Vitreoretinal surgery

## Abstract

**Background:**

To investigate the incidence and clinical characteristics of unexplained visual loss in patients with fovea-sparing rhegmatogenous retinal detachment (RRD) during or after silicone oil (SO) tamponade.

**Methods:**

The medical charts of all patients with macula-on RRDs, who underwent pars-plana-vitrectomy (ppV) with SO tamponade were retrospectively assessed regarding unexplained visual loss (UVL) of ≥ 3 Snellen lines and alterations on optical coherence tomography (OCT) during or after SO tamponade. The clinical data analysed included visual acuity, surgical parameters, OCT images, duration of SO tamponade and the time point of visual decline. Cases with re-detachment or secondary causes of visual loss such as SO emulsification, epiretinal membranes or macular edema were excluded.

**Results:**

Over a 15-year-period, 22 cases with macula-on RRD, which had primarily been treated with ppV and SO tamponade, met the inclusion criteria. In most eyes (*n* = 20; 91%), the RRD was caused by a giant retinal tear (GRT). In 11 of these 22 cases (50%), best-corrected visual acuity (BCVA) had dropped by at least 3 lines for no apparent reason. In these 11 cases, mean preoperative logMAR BCVA was 0.2 (SD 0.13; range 0-0.5), equal to Snellen’s VA of 0.63, and mean postoperative logMAR BCVA 1.0 (SD 0.24; range 0.5–1.3), equal to Snellen’s VA of 0.10. Visual decline occurred about 12 weeks postoperatively (SD 6.2; range 3–20 ) and comprised 8 lines (SD 2.3; range -11 to -4). SO was removed on average 139 (SD 50.0; range 88–271) days after the first ppV. In 9 cases visual decline occurred while the SO was in-situ. In 2 patients, BCVA decline was noted 2 weeks after SO removal. In all eyes, preoperative central foveal thickness (CFT) was 254 μm (SD 24.2), which decreased to 224 μm (SD 29.6) during SO tamponade and increased to 247 μm (SD 29.2) after SO removal, irrespective of the presence of UVL. The mean follow-up time was 20 months (SD 30.6) after SO removal.

**Conclusion:**

UVL after SO tamponade for macula-on RRD is more frequent than expected. The incidence in our case series was 50%. The mechanism of this phenomenon is still unknown. In general, vitreoretinal surgeons should thoroughly question the need for SO tamponade, inform their patients of possible UVL and remove SO as early as possible.

**Trial registration:**

The study was approved by the local ethics committee on 6th of May 2022 (Ethikkommission der Universität Regensburg, Votum 22-2925-104) and was conducted in accordance with the ethical standards of the Declaration of Helsinki.

## Introduction

For decades, silicone oil (SO) has been routinely used as an intraocular tamponade for pars-plana-vitrectomy (ppV) in cases with complex rhegmatogenous retinal detachment (RRD) [1]. In most cases, in which vitreoretinal (VR) surgeons decide to use SO, visual prognosis is limited due to long-standing detachment, macula-off situations, primary proliferative vitreoretinopathy (PVR) or the status after trauma or previous VR surgery. Only few cases with RRD receive a ppV with primary SO tamponade. The initial functional prognosis is relatively good, if the RRD is macula-on and in the absence of PVR or previous ocular events. In these situations, the possible side effects of a SO tamponade have to be considered in detail. SO is a well-established medium for VR surgery because of its long-lasting intraocular tamponade and optical clarity, which allows immediate postoperative assessment. Yet, there a several disadvantages [2]: Apart from well-known SO-related effects such as secondary glaucoma or corneal decompensation, some patients develop unexplained visual loss (UVL) during or after SO tamponade [1–5]. Therefore, we decided to evaluate all our patients with RRD who had received a primary SO tamponade within the past 15 years.

## Methods

We retrospectively analysed all consecutive patients presenting with a macula-on RRD, who had undergone primary ppV with SO tamponade and experienced an UVL of ≥ 3 Snellen lines during or after the SO tamponade between 2007 and 2021. Patients with UVL during or after SO tamponade for macula-on detachment repair were compared to patients with good functional outcome. Medical charts were reviewed by means of standardised data collection including age, sex, ocular history and best corrected visual acuity (BCVA) at the initial visit and at follow-up. Patients' surgical logbooks were evaluated regarding the technique and duration of surgery, the type of SO used, the duration of the procedure, the macular status and any intraoperative abnormalities. We systematically evaluated the postoperative course, the BCVA, the morphological parameters based on spectral-domain optical coherence tomography (OCT) imaging (Spectralis®, Heidelberg Engineering), the duration of the SO tamponade and the time point of SO removal. The OCT scanning protocol comprised a 6-line macular star with a length of 6 mm centred on the fovea. The OCT interpretation included central foveal thickness (CFT) and integrity of the inner and outer retinal layers.

The following inclusion criteria were defined: good preoperative Snellen's VA of ≥ 0.3, RRD with macula-on status and SO as primary tamponade and at least 3 months of follow-up after SO removal. Exclusion criteria were recurrent RRD after primary ppV, PVR grade C or higher, history of trauma or previous intraocular surgery, glaucomatous optic disc changes or other pre-existing ocular diseases affecting visual outcome as well as postoperative complications such as endophthalmitis, epiretinal membranes or outer retinal layer defects, distinct macular edema or subretinal perfluorocarbon remnants.

Statistical analysis was done with SPSS statistics 25 (IBM, USA). Continuous values are presented as means with standard deviation (SD) and categorical variables as frequency counts with percentages. Pre- and postoperative BCVA values were converted to logMAR. Continuous variables were compared using the student's t-test. For categorical variables, the x^2^ test or the Fisher's exact test were used. A *p*-value < 0.05 was considered statistically significant.

## Results

Overall, 22 eyes that had undergone primary ppV with SO tamponade in a macular-on situation with a preoperative BCVA ≥ 0.3, met the inclusion criteria. In 11 of these 22 eyes (50%), a UVL of at least 3 Snellen lines had occurred during or after SO tamponade. The other 11 eyes without UVL were analysed as comparison.

### Preoperative data

Overall, 22 eyes of 12 men and 8 women with a mean age of 52 years at presentation (SD 10.2; range 33–73 years) had undergone surgery for macula-on RRD with primary SO tamponade. One man in the UVL group and one woman in the comparison group had sequential bilateral involvement. The two groups did not statistically differ in basic characteristics such as age, sex, lens status, duration of symptoms before presentation and the time between RRD diagnosis and surgery. On average, 2 quadrants of the retina (SD 0.9) were involved. Posterior vitreous detachment (PVD) was present in 21 of 22 eyes. In most eyes (*n* = 20; 91%), macula-on RRD was caused by a giant retinal tear (GRT). In 2 eyes, SO was used because of multiple retinal holes. The basic preoperative data of the UVL group and the comparison group are listed in Table 1.

**Table 1:** Basic preoperative data of 22 cases with macula-on retinal detachment (UVL = unexplained vision loss, RRD = rhegmatogenous retinal detachment, GRT = giant retinal tear)

	Cases with UVL (*n* = 11)	Cases without UVL (*n* = 11)	*p*-value
Sex	male 9 (82%)	male 4 (36%)	0.080
Age (years)	52 (SD 9.1; range 35–63)	51 (SD 11.5; range 33–73)	0.731
Site	right 5 (46%)	right 6 (54%)	0.670
Lens status	phakic 7 (64%)	phakic 6 (54%)	1.000
Extent of RRD (quadrants)	2 (SD 0.9; range 1–4)	2 (SD 1.01; range 1–4)	0.828
GRT	9 (82%)	11 (100%)	0.478
Duration of symptoms before presentation (days)	7 (SD 8.0; range 1–21)	8 (SD 7.3; range 2–21)	0.722
Time between diagnosis and surgery (days)	1 (SD 0.3; range 1–2)	1 (SD 0.4; range 1–2)	0.582

### Surgical approach

All 22 eyes had been primarily treated with a standard 20 G (until 2010) or a 23 Gauge ppV (from 2011 onwards) with SO tamponade under general anaesthesia. On average, the time between RRD diagnosis and surgery was 1 day (SD 0.4; range 0–2 days). Most phakic eyes (11 out of 14 eyes, 79%) were treated with a combination of ppV and phacoemulsification with posterior chamber intraocular lens implantation (phakovitrectomy). After vitrectomy and drainage of subretinal fluid with perfluorocarbon (PFCL, F-Decalin, Fluoron GmbH, Germany), retinal breaks and tears were treated with cryo- or laser-photocoagulation, or both. Afterwards, PFCL was removed completely and exchanged first for air and afterwards air for SO (Oxane® 5700, Bausch + Lomb). Each groupcontained 1 case with a direct exchange of PFCL for SO to prevent slippage of the retina. The two groups did not statistically differ in cut-suture-time, combination with phacoemulsification, number of laser spots and use of 360° laser and cryocoagulation. Table 2 lists all relevant surgical aspects in detail (see Table 2).

**Table 2:** Surgical data of 22 cases with macula-on retinal detachment (UVL = unexplained vision loss)

	Eyes with UVL(*n* = 11)	Eyes without UVL (*n* = 11)	*p*-value
Cut-suture-time (minutes)	85 (SD 25.5)	71 (SD 25.2)	0.188
Phakovitrectomy	6 (54%)	5 (46%)	1.000
Laser spots	723 (SD 348.1)	776 (SD 359.2)	0.735
360° laser-retinopexy	8 (73%)	6 (54%)	0.659
Cryocoagulation	9 (82%)	11 (100%)	0.476

### Visual acuity

Before surgery, the mean logMAR BCVA had been 0.2 (SD 0.15; range 0–0.5), equivalent to a Snellen's VA of 0.63. The preoperative BCVA did not statistically differ between the two groups (*p* = 0.605). In 11 of the 22 cases (50%), a severe loss of BCVA was noted postoperatively for no apparent reason. The patients in the UVL group noted the deterioration of BCVA on average 12 weeks after surgery (SD 6.2; range 3–20). In 9 cases, the visual decline occurred while the SO was in-situ. 2 patients noted visual decline about 2 weeks after SO removal when the transient air tamponade had dissolved. In the comparison group, 2 eyes had mild loss of BCVA (1–2 lines) due to subtle intraretinal edema or secondary posterior capsule opacification, 3 eyes kept a stable BCVA and the other 6 eyes showed improved BCVA at follow-up. After SO removal, mean logMAR BVCA in the UVL group was 1.0 (SD 0.22; range 0.5–1.3), equivalent to a Snellen's VA of 0.1, and 0.2 (SD 1.72; range 0–0.5) in the comparison group, equivalent to a Snellen's VA of 0.63. The two groups showed a statistically significant difference in the final logMAR BCVA (*p* < 0.05) and in the mean difference in BCVA lines (*p* < 0.05). On average, the follow-up period comprised 20 months (SD 30.6) after SO removal. Table 3 shows detailed BCVA values for both groups before surgery, during SO tamponade and after SO removal.

**Table 3:** Functional outcome of 22 cases with macula-on retinal detachment (UVL = unexplained vision loss, BCVA = best corrected visual acuity, SO = silicone oil)

	UVL group (*n* = 11)	Comparison group (*n* = 11)	*p*-value
logMAR BCVA before SO tamponade	0.2 (SD 0.13; range 0–0.5)	0.3 (SD 0.19; range 0.1–0.5)	0.605
logMAR BCVA during SO tamponade	0.6 (SD 0.30; range 0.2–1.0)	0.4 (SD 0.16; range 0.2–0.7)	0.148
logMAR BCVA after SO tamponade	1.0 (SD 0.24; range 0.5–1.3)	0.3 (SD 0.12; range 0–0.5)	< 0.05
Δ BCVA (lines)	-8 (SD 2.3; range -11 to -4)	0.3 (SD 1.63; range -2 to + 2)	< 0.05

### Morphological parameters on OCT imaging

**Fig. 1:**
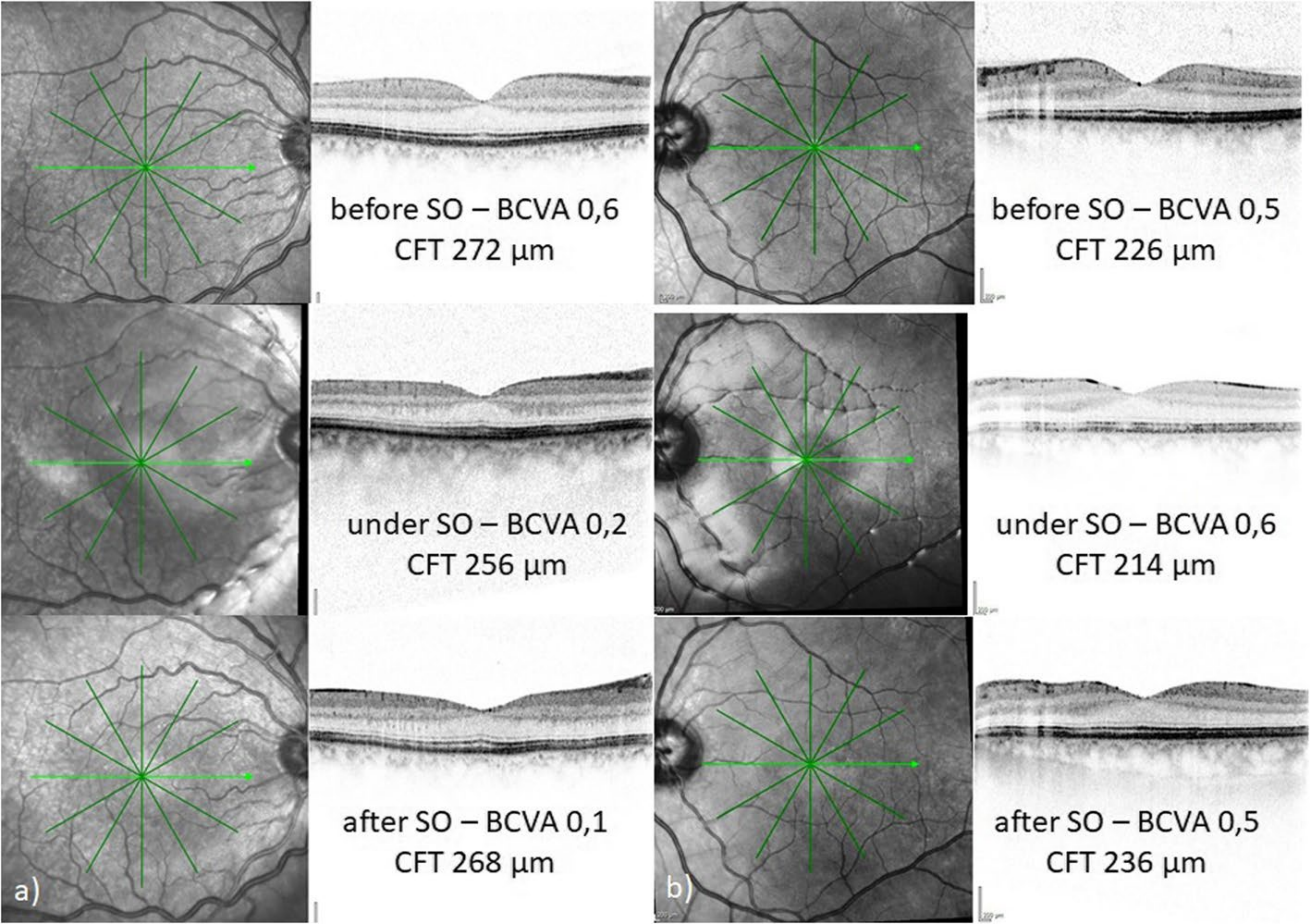
Follow-up OCT images of eyes before, during and after silicone oil (SO) tamponade, showing changes in central foveal thickness (CFT). [BCVA = best corrected visual acuity, CFT = central foveal thickness]. **a** Right eye of a 47-year-old man with 5 months of SO tamponade, who noted severe reduction of best corrected visual acuity (BCVA) after 4 months. **b** Left eye of a 41-year-old man with 3 months of SO tamponade without visual loss

Due to the urgency of macula-on RRD treatment, only 4 eyes in each group had received a preoperative OCT scan. Postoperative OCT data during SO tamponade were obtained from 16 eyes. Mean preoperative CFT was 254 µm (SD 24.2; range 220–289 µm), which dropped to 224 µm (SD 29.6; range 151–256 µm) during SO tamponade and increased again to 247 µm (SD 29.2; range 199–283 µm) after SO removal. CFT reduction during SO tamponade was statistically significant for all eyes (*p* = 0.007), but was not significant in either group (UVL group: *p* = 0.083; comparison group: *p* = 0.115). The increase in CFT after SO removal was statistically significant for all eyes and in both groups (all: *p* = 0.001; UVL group: *p* = 0.004; comparison group *p* = 0.032). The CFT did not statistically differ between the two groups, neither before, during or after SO tamponade. After SO removal, mean CFT resembled that of the fellow eye. Table 4 gives a detailed overview of OCT parameters (see Table 4). Figure 1 shows examples of CFT development before, during and after SO tamponade of one patient of each group (see Fig. 1).

**Table 4:** Pre- and postoperative OCT parameters in the UVL and comparison group (UVL = unexplained vision loss, CFT = central foveal thickness, SO = silicone oil)

	UVL group	Comparison group	*p*-value
CFT before SO tamponade [mm]	*n* = 4 244 (SD 26.1)	*n* = 4 264 (SD 20.5)	0.726
CFT during SO tamponade [µm]	*n* = 9 227 (SD 27.1)	*n* = 7 221 (SD 34.6)	0.282
CFT after SO tamponade [µm]	*n* = 9 241 (SD 26.8)	*n* = 7 244 (SD 31.7)	0.630
CFT of fellow eye after SO tamponade [µm]	*n* = 3 246 (SD 21.8)	*n* = 7 255 (SD 45.7)	0.534

**Fig. 2:**
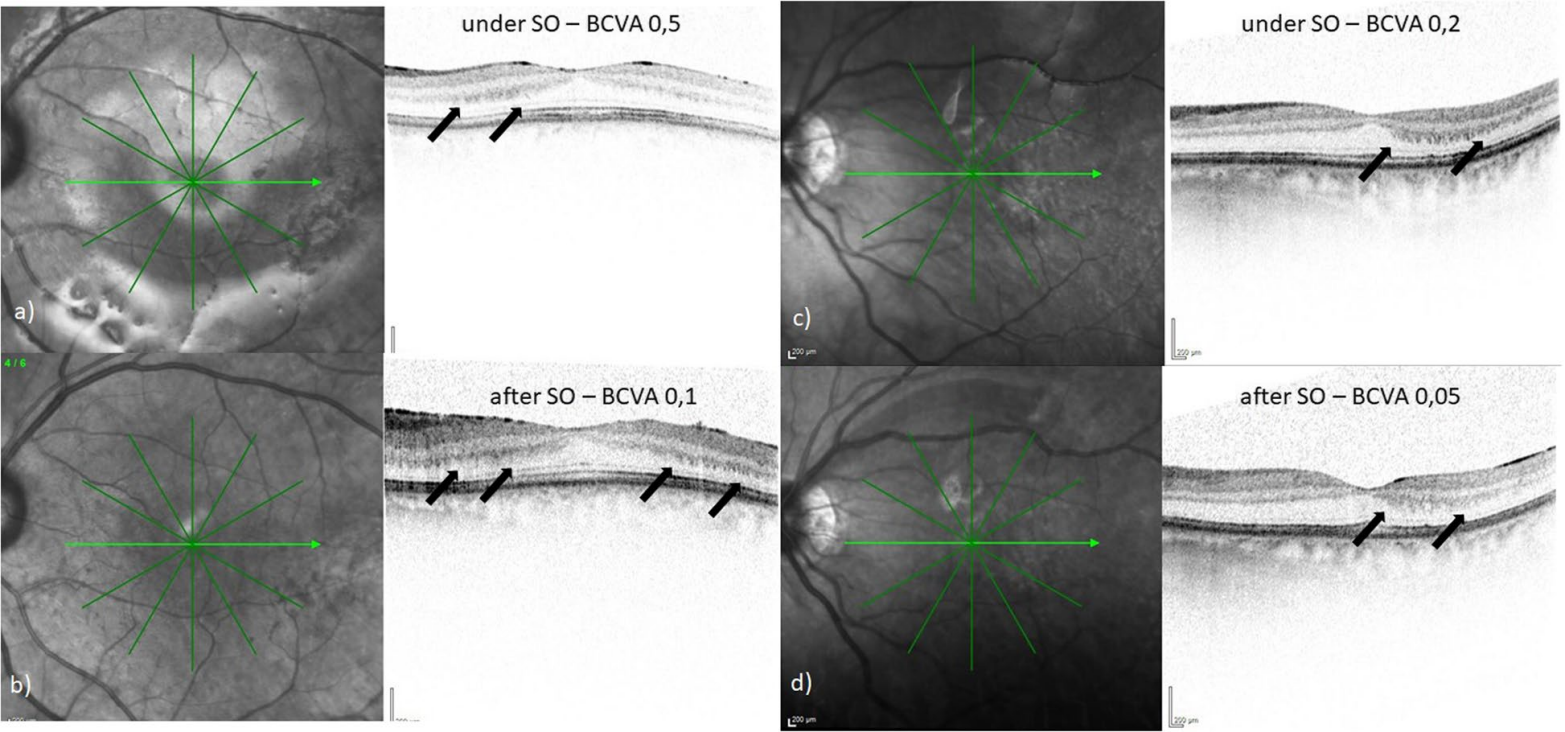
Follow-up OCT images of 2 eyes with unexplained visual loss (UVL) under silicone oil (SO) tamponade, showing alterations at the level of the outer plexiform layer (OPL) corresponding to higher reflectivity of Henle’s fibre layer. [BCVA = best corrected visual acuity] a/b) Left eye of a 63-year-old woman with UVL after 4 months, duration of SO tamponade of 5 months and OPL changes ( →) during (**a**) and after (**b**) SO tamponade. c/d) Left eye of a 50-year-old man with UVL after 4,5 months, duration of SO tamponade of 4 months and OPL alterations ( →) during (**c**) und after (**d**) SO tamponade

**Fig. 3:**
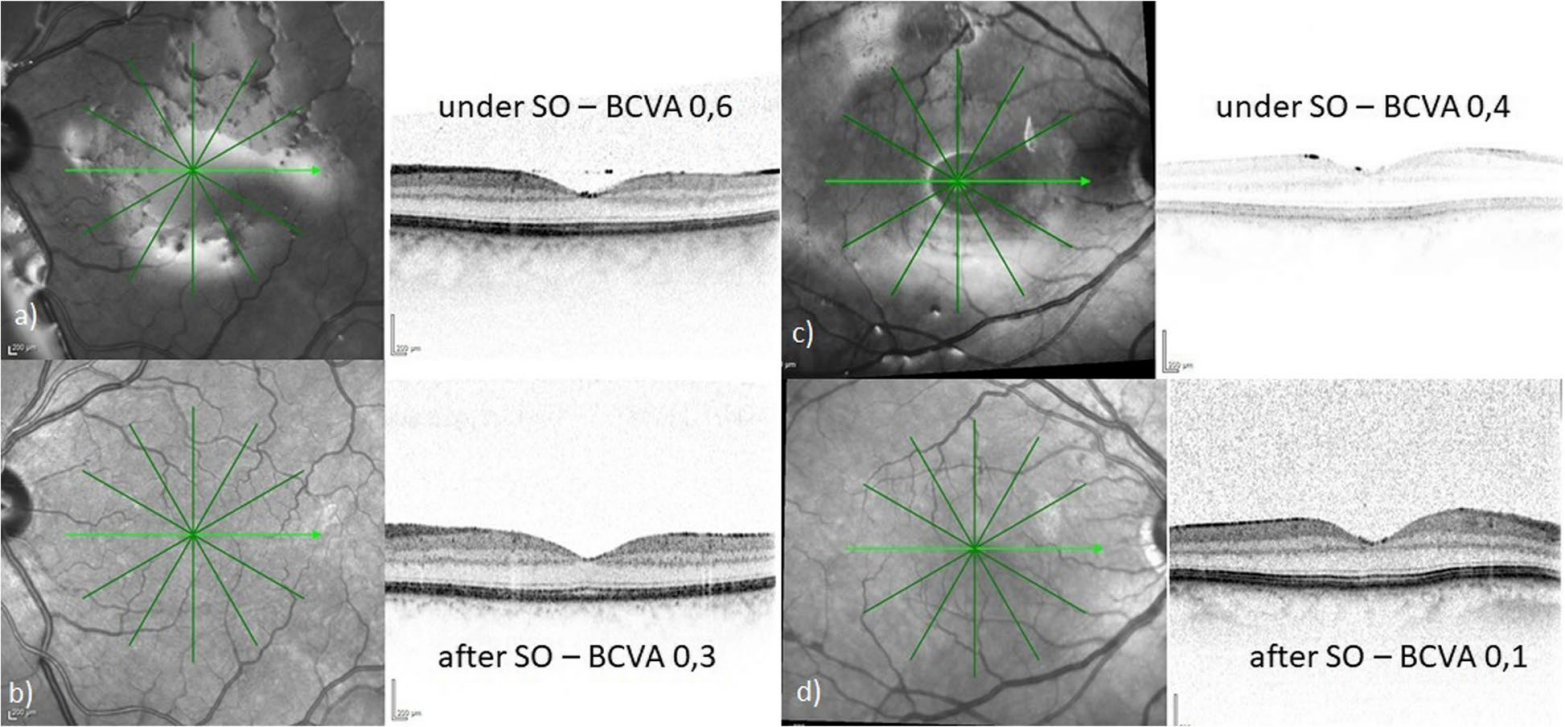
Follow-up OCT images of eyes during and after silicone oil (SO) tamponade in 2 patients with unexplained visual loss (UVL). Apart from the SO reflexes, OCT scans show no specific alterations of the inner or outer retinal layers. [BCVA = best corrected visual acuity] a/b) Left eye of a 58-year-old man with 4 months of SO tamponade during (**a**) and after SO tamponade (**b**). c/d) Right eye of 59-year-old man with 3 months of SO tamponade during (**c**) and after SO tamponade (**d**)

Because automated segmentation of the individual retinal layers on OCT images was not available at our unit, we analysed other qualitative aspects such as the integrity of the inner and outer retinal layers on all existing OCT scans during SO tamponade and after SO removal. In the UVL group, 7 of 11 eyes showed mild changes at the level of the outer plexiform layer (OPL) during and/or after SO tamponade (see Fig. 2). These OCT alterations mirror artefacts of the higher reflective Henle’s fibre layer, possibly caused by a smaller postoperative pupil size and eccentricity of the entry beam [6]. No alterations in the ellipsoid zone and Bruch’s-membrane complex were seen (see Fig. 3).

### Postoperative course

The postoperative intraocular pressure (IOP) during SO tamponade ranged on average from a minimum IOP of 12 mmHg (SD 12.2; range 2–18 mmHg) to a maximum IOP of 22 mmHg (SD 8.7; range 2–42 mmHg). The two groups did not differ in minimum and maximum IOP under SO tamponade. The second ppV with removal of the SO was done on average after 122 days (SD 48.0). In the UVL group, SO was removed about 2.5 weeks later than in the comparison group. However, this difference in the duration of SO tamponade was not statistically significant (*p* = 0.240). Table 5 lists data of the postoperative course (see Table 5).

**Table 5:** Postoperative data of 22 cases with macula-on retinal detachment (UVL = unexplained vision loss, SO = silicone oil)

	UVL group (*n* = 11)	Comparison group (*n* = 11)	*p*-value
Duration of SO tamponade (days)	139 (SD 50.0; range 88–271)	122 (SD 48.0; range 55–185)	0.240
Minimum IOP under SO (mmHg)	13 (SD 2.8; range 7–18)	11 (SD 3.8; range 2–16)	0.136
Maximum IOP under SO (mmHg)	24 (SD 5.6; range 18–37)	18 (SD 8.3; range 2–32)	0.450

## Discussion

Severe visual loss in eyes with good preoperative BCVA and favourable functional prognosis due to macula-on-status is a devastating adverse event for the patient, especially if there is no explanation for this phenomenon. Here, we report a retrospective 15-year analysis of 11 cases with UVL during or after SO tamponade. The limitations of our study are its retrospective design, the small sample size, the lack of automated OCT layer segmentation and optic nerve head status (e. g. optic disc pallor and peripapillary nerve fibre layer thickness). Moreover, all procedures were done under general anaesthesia, which is not common practice in other parts of the world. Following our inclusion criteria of macula-on RRDs with primary SO tamponade, mostly GRT-associated detachments were analysed. Therefore, our results might not be transferred to macula-on RRDs in general. However, we had also cases with UVL in SO-filled eyes without GRT. In view of the rare use of primary SO for macula-on RRDs, larger prospective trials are not likely.

Our analysis yielded a rate of 50% of eyes that developed UVL during or after SO tamponade without any recovery, even after a mean follow-up time of 20 months (SD 30.6) after SO removal. Other authors reported rates of 3–11% for all RRDs with SO including macula-off situations [3, 4, 7] and rates of 20–50% for eyes with macula-on-detachment [3, 5, 8, 9]. In view of the literature and our own results, we estimate the incidence of UVL during or after SO tamponade for eyes with macula-on status and good baseline prognosis to be approximately 50%.

The two groups did not differ in baseline characteristics. The UVL group had a noticeable higher but not statistically significant proportion of male patients than the comparison group (82% vs. 36%). Other studies reported similar baseline data with an equal distribution of male patients in the UVL group and the comparison group of about 60% [2, 4, 8]. Our UVL group showed severely reduced mean logMAR BCVA of 1.0 (equalling 0.1 of Snellen's VA), while the BCVA in the comparison group had remained stable. Other authors reported similar functional results with a final logMAR BCVA after SO removal between 0.78 and 1.00 [2, 3, 5]

The pathophysiology of UVL during or after SO tamponade is still unknown. Possible reasons may be SO-related structural changes of the retina with subsequent macular dysfunction, SO emulsification, SO-related phototoxicity and dissolution of fat-soluble lutein and zeaxanthin, altered homoeostasis of the vitreous cavity and SO-related affections of the optic nerve resulting in atrophy of the retinal nerve fibre layer (RNFL) [1, 4]. Gonvers et al. noted retinal lesions in the OPL of rabbit eyes 6 weeks after SO injection [10]. Papp et al. found a reduction of 89% in myelinated optic nerve fibres in rabbit eyes after 1 year of SO tamponade [11]. In enucleated human eyes, intra- and extracellular SO deposits within the retina were seen in electron microscopy [1, 12, 13], but there are also cases with SO deposits within the RNFL without any visual loss [9]. In some rare cases, MRI has shown retrograde migration of SO bubbles along the optic nerve into the brain, but these findings could not be confirmed in serial MRI examinations [14, 15]. Some authors hypothesised that altered potassium levels may affect Müller cells in SO-filled eyes, whereas Scheerlinck et al. measured a decrease in magnesium concentration in SO-filled eyes compared to vitreous humour without any change in potassium levels [7, 16, 17]. However, the degeneration of Müller cells supported by OCT findings of thinning of the inner retinal layers is a potential causative factor that is gaining support [16].

Regarding OCT imaging, several authors reported changes in retinal layer thickness and structural alterations on OCT scans in patients with SO-related visual loss [2, 5, 9, 18, 19]. In our study, we found reduced CFT during SO tamponade independent of the presence of UVL. In a case series conducted by Tode et al., automated OCT segmentation showed thinning of the inner retinal layers incases with UVL under SO [18]. Purtskhvanidze et al. found an overall thinning of inner retinal layers in SO-filled eyes compared to eyes with gas tamponade [19]. Christensen et al. also reported also a significant thinning of the inner retinal layers in SO-filled eyes compared to gas-operated eyes but no association of reduced CFT and presence of UVL was seen [9]. A meta-analysis by Ghanbari et al. showed significantly reduced CFT in SO-filled eyes compared to eyes with gas tamponade but no significant difference in CFT between eyes after SO removal and fellow eyes [20]. Concordantly, Rabina et al. noted a transient decrease in CFT in a series of 41 eyes with stable VA during SO presence and a regression to fellow eye CFT values after SO removal [21]. The findings by Rabina et.al. resemble the results of our study, in which the CFT changed in all eyes during SO tamponade, irrespective of the occurrence of UVL. Therefore, we do not think that CFT measurement alone is helpful in identifying patients at risk of UVL. Separate measurement of the inner retinal layers may be more specific.

No relevant differences were found in surgical parameters between our UVL cases and the comparison group. The only influenceable factor that varied between the two groups was the duration of SO tamponade (139 ± 50.0 days in UVL cases vs. 122 ± 48.0 days in cases without UVL). In our cohort, this difference was not statistically significant, but other authors identified the duration of SO tamponade as a risk factor for UVL [4, 5]. Scheerlinck et al. reported a longer duration of 5 weeks of SO tamponade in eyes with UVL than in eyes without UVL, whereas Roca et al. assessed a difference of about 5 months between cases with UVL compared to cases without UVL. In concordance with our results, UVL had occurred during SO tamponade or after SO removal [5, 9]. The minimum duration of SO presence in cases with UVL was 12 weeks [5], which matches our results of a minimal range of 88 days of SO tamponade in UVL cases.

In summary, we present detailed clinical and surgical data of cases with UVL during or after SO tamponade, which were compared to cases with similar baseline characteristic without UVL. So far, no alternative endotamponade is available that could completely replace SO. PFCL as a short-term tamponade is sometimes discussed as an alternative option [22], but here our experience is limited. Long-lasting intraocular gas is an option in some macula-on-situations with GRTs when no slippage of the break is seen after PFCL-air-exchange. Compared to SO tamponade in macula-on cases with GRT, gas tamponade has a significantly lower rate of UVL [8, 9] but no significantly different re-detachment rate [8]. However, cases with slippage of the GRT may require SO to prevent anatomical failure.

The only factor differing between the groups that can be influenced by the VR surgeon, was the duration of SO tamponade; hence, we propose the following: 1. to question the need for SO in every situation, 2. to inform the patient about possible visual loss, 3. to closely follow-up patients with SO tamponade in macula-on situations and 4. to keep the duration of SO tamponade in macula-on detachments as short as possible.

## Data Availability

The data that support the findings of this study are available from teresa.barth@ukr.de. Restrictions apply to the availability of these data. Because of the low sample size, conclusions could be drawn just by age and the year of surgery, and patients could be identified. Data are however available from the authors upon reasonable request and with permission of the local ethics committee.

## Abbreviations

BCVA: Best corrected visual acuity
CFT: Central foveal thickness
GCL: Ganglion cell layer
GRT: Giant retinal tear
IPL: Inner plexiform layer
RRD: Rhegmatogenous retinal detachment
OCT: Optical coherence tomography
OPL: Outer plexiform layer
PFCL: Perfluorocarbon
PVD: Posterior vitreous detachment
PVR: Proliferative vitreoretinopathy
SO: Silicone oil
UVL: Unexplained vision loss
VA: Visual acuity
VR: Vitreoretinal
