# Correction to: Unexplained visual loss after primary pars-plana-vitrectomy with silicone oil tamponade in fovea-sparing retinal detachment

**DOI:** 10.1186/s12886-023-02861-0

**Published:** 2023-03-16

**Authors:** T. Barth, H. Helbig, D. Maerker, M.-A. Gamulescu, V. Radeck

**Affiliations:** Department of Ophthalmology, University Medical Centre Regensburg, Franz-Josef-Strauß-Allee 11, 93053 Regensburg, Germany

**Correction: BMC Ophthalmol 23, 75 (2023)**

10.1186/s12886-023-02823-6

Following publication of this article [1], it has been brought to our attention that the author name of T. Barth was duplicated.

The author group has been updated above and the original article has been corrected.
